# Canonical and Non-Canonical Aspects of JAK–STAT Signaling: Lessons from Interferons for Cytokine Responses

**DOI:** 10.3389/fimmu.2017.00029

**Published:** 2017-01-26

**Authors:** Andrea Majoros, Ekaterini Platanitis, Elisabeth Kernbauer-Hölzl, Felix Rosebrock, Mathias Müller, Thomas Decker

**Affiliations:** ^1^Department of Microbiology, Immunobiology and Genetics, Max F. Perutz Laboratories, University of Vienna, Vienna, Austria; ^2^Institute of Animal Breeding and Genetics, University of Veterinary Medicine Vienna, Vienna, Austria

**Keywords:** signal transduction, JAK–STAT, non-canonical, interferon, innate immunity

## Abstract

Janus kinase (JAK)–signal transducer and activator of transcription (STAT) signal transduction mediates cytokine responses. Canonical signaling is based on STAT tyrosine phosphorylation by activated JAKs. Downstream of interferon (IFN) receptors, activated JAKs cause the formation of the transcription factors IFN-stimulated gene factor 3 (ISGF3), a heterotrimer of STAT1, STAT2 and interferon regulatory factor 9 (IRF9) subunits, and gamma interferon-activated factor (GAF), a STAT1 homodimer. In recent years, several deviations from this paradigm were reported. These include kinase-independent JAK functions as well as extra- and intranuclear activities of U-STATs without phosphotyrosines. Additionally, transcriptional control by STAT complexes resembling neither GAF nor ISGF3 contributes to transcriptome changes in IFN-treated cells. Our review summarizes the contribution of non-canonical JAK–STAT signaling to the innate antimicrobial immunity imparted by IFN. Moreover, we touch upon functions of IFN pathway proteins beyond the IFN response. These include metabolic functions of IRF9 as well as the regulation of natural killer cell activity by kinase-dead TYK2 and different phosphorylation isoforms of STAT1.

## Introduction

Since their discovery in the late 1950s (1), interferons (IFNs) have been assigned various functions that extend far beyond the initially observed antiviral activity. Three families of IFNs have been described and are known as type I (IFN-I, mainly IFNα/β), type II (IFN-II or IFNγ), and type III (IFN-III or IFNλ). In canonical IFN signaling, all types of IFNs produce a transcriptionally active signal transducer and activator of transcription 1 (STAT1) through receptor-bound Janus kinase (JAK)-mediated phosphorylation of tyrosine (Y) 701. The IFNγ receptor employs JAK1 and JAK2 to phosphorylate exclusively STAT1, causing its homodimerization. STAT1 dimers, also called gamma interferon-activated factor (GAF), translocate to the nucleus and promote gene expression by binding to gamma interferon-activated site (GAS) of interferon-stimulated genes (ISG). On the other hand, stimulation with IFN-I or IFN-III leads to TYK2- and JAK1-mediated phosphorylation of STAT1 and STAT2. After forming heterodimers, these two proteins associate with interferon regulatory factor 9 (IRF9) to form a transcriptionally active IFN-stimulated gene factor 3 (ISGF3) that controls gene expression by binding to interferon-stimulated response elements (ISRE) in a different set of ISGs (2, 3). Although a large majority of IFN-induced gene expression is mediated by canonical pathways, it has become clear that the components of these pathways are able to exert non-canonical activity: tyrosine kinase-independent action of JAKs, transcriptional complexes other than ISGF3 and GAF, and pathways building on U-STATs that are not phosphorylated on tyrosine. This review will focus on these non-canonical functions of JAK–STAT signaling components.

## IFNs and Their Role in Resistance to Viruses and Bacteria

As major components of the innate immune system against viral infections, all type I interferons (IFN-I) stimulate cell-autonomous antiviral activity (4). In addition, they increase cellular immunity through contributions to natural killer (NK) and T cell activation (5). IFN-I act as modulators of cellular immunity by selectively enhancing clonal expansion and survival of CD8+ T cells (6), directing the immune response toward Th1-dominace (7) and activating NK cells (8).

In the context of antibacterial defense, genes activated by IFN-I enhance inflammation and the death of infected cells (9). In addition, they impact on cells at the interface of the innate and adaptive immune systems, such as macrophages and dendritic cells, to increase antigen presentation and trigger the adaptive response (10). Immunostimulatory activities of IFN-I also contribute to immunosurveillance against cancer (11). On the other hand, their proinflammatory activity renders them driving forces behind a group of interferonopathies, such as the Aicardi-Goutieres syndrome (12, 13).

While IFN-I are generally protective against viral infections, they can be both friend and foe in the defense of bacterial pathogens (14, 15). For example, IFN-I exert protective effects in the case of *Chlamydia pneumoniae* (16), *Legionella pneumophila* (17), *Salmonella typhimurium* (18), and both group A and group B *Streptococcus* infections (19–21). However, in infections with *Listeria monocytogenes, Francisella tularensis*, and *Mycobacterium tuberculosis*, production of IFN-I is associated with decreased innate immunity (22–27).

Major target cells of IFNγ are macrophages and T cells. Many of the genes induced by IFNγ are transcription factors, which amplify the transcriptional response and, as in Th cells, influence cell differentiation (28–30). IFNγ is particularly important in macrophage biology, where it provides cell-autonomous antimicrobial activity through the upregulation of microbicidal gene products (31). Further, impact of IFNγ on macrophage activation results from its ability to synergize with or to antagonize the effects of different cytokines, growth factors, and pathogen-associated molecular pattern-signaling pathways (e.g., TNFα, IL-4, CSF-1, IFNα/β, LPS, and CpG DNA). Through these mechanisms, IFNγ activates macrophages to express antimicrobial and antitumor effects. It upregulates chemokines and adhesion molecules, directing cells to the sites of inflammation. In the adaptive immunity, IFNγ plays an important role in Th1 responses, repressing the development of Th2 and Th17 T cell responses (30) and acting directly on B cells to promote class switching from IgG2 to IgG3 (32). Mice and humans deficient in IFNγ or IFNGR1 show a decrease in natural resistance to bacterial, parasitic, and viral infections (33–35). Mice and cells lacking IFNγ display compromised tumor rejection, underlining its importance in tumor surveillance (11).

Discovered in the year 2003, IFN-III, better known as IFNλ, are the most recently described members of the IFN family (36). Although signaling through a different receptor complex with IFNλR/IL10R2 chains, IFNλ also stimulate formation of the ISGF3 complex. Given the similarities between the IFN-I and IFNλ signaling pathways, some of the non-canonical signals described below for IFN-I may apply to IFNλ as well. While IFNλ produce similar biological changes of their target cells as IFN-I, including antiviral, antiproliferative, and antitumor activity, the key to different organismic responses to IFN-III lies in their receptor distribution, which is prevalent on cells of epithelial origin (37). In line with that, defects in IFN-III production or signaling cause reduced innate immunity to viral pathogens replicating in epithelia of the lung and the gut. Mice lacking the IFN-I receptor are resistant to all IFN-I subtypes, but retain their sensitivity to IFN-III (38). However, studies performed in IFNAR1^−/−^, IL-28Rα^−/−^, and IFNAR1/IL-28Rα double knockout mice show that, in primary airway epithelia, upon influenza infection, IFN-I and IFN-III mediate parallel amplification loops that lead to the induction of fully overlapping groups of ISGs (39).

## Kinase-Independent JAK Activity

Janus kinases are non-receptor tyrosine kinases which have essential roles in cytokine and growth factor signaling (40, 41). There are four different JAKs (JAK1, JAK2, JAK3, and TYK2) that cross-phosphorylate and activate each other when ligand-associated cytokine receptor chains come in close proximity. Canonical, i.e., kinase-dependent functions of JAKs include tyrosine phosphorylation of receptor chains and of STATs at a single tyrosine residue near the C-terminal end. STAT phosphorylation is thought to require SH2 domain-mediated docking to the modified receptor chains, consistent with impaired phosphorylation at mutant receptors lacking the critical tyrosine for JAK-mediated phosphorylation (2).

Recently, reports studying kinase-inactive mutants of TYK2 and JAK2 suggest that these proteins exert functions not requiring their kinase activity and have important non-canonical roles. Elegant studies in mouse models with kinase-dead enzymes allow the demonstration of kinase-independent functions under physiologic conditions and complement studies in human and murine cell lines. Furthermore, description of naturally occurring mutations in JAKs in the human population broadens our understanding of the multifaceted role of JAKs in health and disease.

### TYK2

TYK2 is involved in a large number of cytokine signaling cascades as it associates with the IFN-I (IFNAR), IL-12Rβ1, IL-10R2, gp130, and IL13α1 receptor (42). Early work in human cell lines has demonstrated the important function of TYK2 in IFN-I signaling (43–46), as human cells fail to respond to IFNα in absence of TYK2. Furthermore, such cells display reduced IFNAR expression at the cell surface. Interestingly, this is a consequence of a non-canonical role of TYK2. The scaffold TYK2, but not its kinase or pseudokinase domains are needed for surface expression of IFNAR in human 11,1 cells (47). Specifically, TYK2 masks a tyrosine-based motif found in IFNAR, thereby shielding the receptor from endocytosis and preventing the binding of an enzyme (AP2), which leads to ubiquitin-dependent internalization (48). In this context, TYK2-receptor association requires neither ligand nor ubiquitination. Preventing receptor degradation is a species-specific TYK2 activity, consistent with the lack of the Tyr-based motif in the murine IFNAR. However, TYK2 is not essential for IFN-I signaling in murine cells, and residual IFN-I activity is detected in the absence of this JAK (49, 50).

Mice with a targeted mutation of a critical lysine residue in the ATP-binding pocket of the TYK2 kinase domain (K923E) express a kinase-dead enzyme (51). Kinase-dead TYK2 shows a strongly reduced half-life owing to increased turnover *via* autophagosomal degradation. The reduced levels of TYK2K923E do not increase IFN-induced STAT activation above the level seen in the Tyk2^−/−^ animals. Consistently, TYK2^K923E^ mice are more susceptible to viral infections, comparable to Tyk2^−/−^ mice. In contrast, TYK2^K923E^ expression partially rescued the defect in natural killer cell (NK-cell) maturation and tumor killing that accompanies TYK2 deficiency (Table 1). At present, this first observation of a kinase-independent *in vivo* function of TYK2 cannot be assigned to a defined NK signaling pathway. Other attributes of activated NK cells like IL-12 synthesis or activating receptor-stimulated production of IFNγ rely on TYK2 kinase activity (52).

**Table 1:** **An overview of non-canonical Janus kinase (JAK)–signal transducer and activator of transcription (STAT) signaling by components of interferon (IFN) pathways**.

Genotype	Non-canonical STAT complexes	Function
WT overexpressing IFN-stimulated gene factor 3 (ISGF3) subunits	Unphosphorylated ISGF3 complex	Prolonged IFN-I responses, resistance to DNA damage
WT expressing high levels of STAT2	pYSTAT1/U-STAT2	Inhibition of STAT1 nuclear translocation, quenching of IFNγ response
STAT1^Y701F^	STAT1Y701F/STAT2/?	Inhibition of STAT2 nuclear translocation, quenching of type I IFN response
	STAT1Y701F/?	Natural killer (NK) cytotoxicity
STAT1^S727A^	STAT1S727A dimer	Reduction of IFNγ response
	STAT1S727A/?	NK cytotoxicity
STAT1^−/−^	STAT2/interferon regulatory factor 9 (IRF9)	Flavivirus and *Legionella pneumophila* resistance
STAT2^−/−^	STAT1/IRF9	IFNγ—colitis, IFN-I—*Legionella pneumophila* resistance
TYK2^K923E^	–	NK cytotoxicity, mitochondrial respiration, IFNAR stability (in humans)
JAK2^KD^	–	IFN-gamma receptor stability, residual IFNγ response

*“?” represents unknown interactors*.

Ligation of the IFNAR causes TYK2-dependent activation of the phosphoinositide 3 kinase (PI3K) pathway. Reportedly, this occurs without a need for TYK2 kinase activity (45). Moreover, in murine pro B cells, a part of TYK2’s functions in mitochondrial respiration is retained in absence of its kinase activity (53). The cells show a drastic defect in basal oxygen consumption and steady-state cellular ATP levels in the absence of TYK2, which can be reversed after transfection of wild-type or a kinase-dead mutant of TYK2 into these cells. In contrast, the kinase activity of TYK2 is required for other functions of mitochondria like complex I-mediated respiration and the induction of apoptosis after IFNβ treatment. TYK2 has also been linked to the energy expenditure of cells, to the regulation of lipid metabolism, differentiation of brown adipose tissue, and obesity (54–56). In human obese patients and obese mice, decreased TYK2 levels are associated with increased obesity. This effect is regulated *via* Stat3 signaling and prolonged stability of the transcriptional coactivator PRDM16, a master regulator of brown adipose tissue (55). The interaction of TYK2 with STAT3 is most probably a non-canonical event, as tyrosine phosphorylation of STAT3 does not require TYK2. Therefore, the kinase must provide another kind of mechanistic input, either indirectly through other pathways or through another modification of STAT3.

The Tyk2 gene displays many different SNPs in the human population, and GWAS studies have linked mutations in Tyk2 to autoimmune diseases like systemic lupus erythematosus, multiple sclerosis, Crohn’s disease, psoriasis, type I diabetes, endometriosis-related infertility, primary biliary cirrhosis, and rheumatoid arthritis (22–25, 57–68). The first patient described with Tyk2 mutation suffered from hyper-IgE syndrome (HIES) and presented with viral, bacterial, and mycobacterial infections. The role of TYK2 in several cytokine signaling pathways leads to these diverse susceptibilities, explained in part by a bias toward Th2 immunity and defective IL-12 and IFN-I pathways (26). Complementing this study, Kreins et al. described seven different patients from four different ethnic backgrounds with different mutations in Tyk2 (27). All patients examined so far had mutations, deletions, or substitutions in the Tyk2 gene which ultimately led to a premature stop codon and no expression of Tyk2 protein. This group of patients did not present HIES. However, similar to the first patient, they suffered from widespread mycobacterial and viral infections. Using microarray analysis, Kreins et al. demonstrated that, similar to TYK2-deficient mice, responses of the patient’s cells to various cytokines like IL-12, IFN-I, IL-23, and IL-10 are greatly reduced, but not completely abolished. This observation, made by the use of a sensitive detection method, might resolve the apparent discrepancy regarding the partial or absolute requirement of TYK2 for cytokine signaling in murine versus human cells. Another study described disease-associated human TYK2 variants, which are catalytically impaired, but able to rescue signaling in response to IFN-I, IL-6, and IL-10 *in vitro* (69). The authors proposed a model for receptors associating with more than one JAK according to which only one JAK needs to be catalytically active in order to convey signals, as long as the second JAK functions as a scaffold.

### JAK2

JAK2 is involved in many biological processes, including the growth control, survival, and differentiation particularly of hematopoietic cells. Accordingly, the widespread use of this kinase is reflected by the embryonic lethality of homozygous deletion (70, 71). The kinase activity of JAK2 is also implicated in various lymphoid and myeloid leukemias in which chromosomal translocation generates a hyperactive kinase (72–74). A scaffold function of JAK2 is suggested by the finding that the N-terminal domain of JAK2 alone is sufficient to enhance surface expression of the Epo receptor (EpoR) (75). Like Tyk2, mice expressing kinase-dead JAK2 were generated and resulted in the discovery of kinase-independent functions. Frenzel et al. generated a mouse model expressing a dominant-negative, kinase-inactive JAK2 by mutating residues in the C-terminal kinase domain (W1038G, E1046R) (76). This mouse mimics the complete loss of JAK2 as homozygous embryos die *in utero*. Heterozygous mice containing this dominant-negative form of Jak2 did not show any hematopoietic abnormalities, and it seems that one intact copy of Jak2 can compensate for the loss of kinase activity of the other, inactive form. Keil et al. generated mice with inactive JAK2 by mutation of the activation loop tyrosines [JAK2^YY1007/1008FF^—(77)]. Similar to the mouse described by Frenzel et al., homozygous JAK2^YY1007/1008FF^ alleles caused embryonic lethality and defective EpoR signaling, whereas heterozygous mice appeared phenotypically normal. However, the study revealed a kinase-independent scaffolding function of JAK2 for the heteromeric IFN-gamma receptor (IFNGR) complex. Importantly, JAK2^YY1007/1008FF^ mediated the cell surface expression of the IFNGR indistinguishable from wild-type JAK2. Likewise, the recruitment of JAK1 to the receptor was normal. Contrasting Jak2-deficient cells, JAK1 alone was able to partly compensate for the loss of JAK2 signaling by phosphorylating STAT1 and inducing genes in response to IFNγ.

## STAT1-Independent Resistance to Viruses and Bacteria—The Role of STAT2 and IRF9

Experiments performed in cells and mice lacking functional STAT1 revealed its indispensable role in the IFN signaling pathway. To date, mice lacking STAT1 have been challenged with at least 27 different pathogens and proved to be highly susceptible to most of the viruses, with exception of (+) single-strand RNA dengue virus (DENV) and the (−) single-strand RNA measles virus (MV). These mice are also highly susceptible to intracellular bacteria, such as *Listeria monocytogenes* and *Mycobacterium tuberculosis*, and parasites such as *Toxoplasma gondii* and *Leishmania major* (78). Patients with complete autosomal recessive STAT1 deficiency succumb to lethal mycobacterial and viral infections. Heterozygous autosomal recessive STAT1 deficiency also causes impaired IFN responses, but with milder clinical symptoms and more positive prognosis. Patients with autosomal dominant STAT1 loss-of-function mutations suffer from mycobacterial diseases and show an impaired response to IFNγ and IL-27 (78, 79). However, the majority of patients with inborn errors of STAT1 show autosomal dominant gain-of-function mutations leading to an unexpectedly broad clinical phenotype, including mucocutaneous candidiasis and autoimmune disorders (80, 81). In addition, some heterozygous *de novo* acquired mutations of the Stat1 gene, affecting coiled-coil and DNA-binding domains, can be associated with progressive combined immunodeficiency. This kind of Stat1 mutations lead to reduction in overall Stat1 expression and signaling responses and are ultimately fatal due to overwhelming infections and inflammation (82).

**Figure 1:**
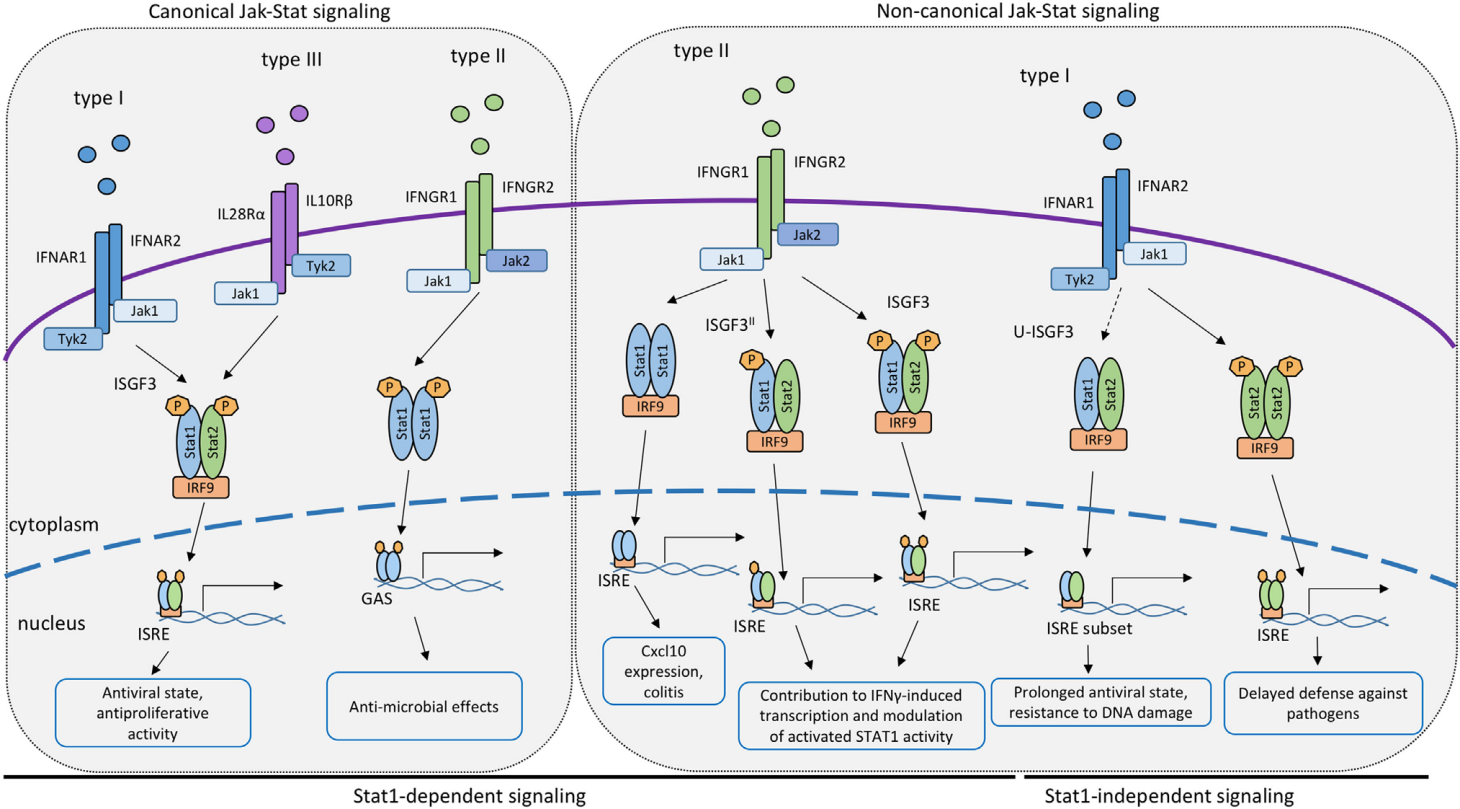
**Canonical and non-canonical STAT signaling by the IFN receptors**. Proposed roles of STAT or STAT/interferon regulatory factor (IRF9) complexes participating in signal transduction and transcriptional activation by the receptors of IFN-I, IFN-II or IFN-III. Complexes containing IRF9 associate with interferon-stimulated response elements promoter sequences whereas dimerized STAT1 binds to GAS elements.

While these findings emphasize the critical importance of STAT1 in IFN responses, studies in mice support the idea that residual IFN-dependent activity against some pathogens remains in absence of STAT1. Initial evidence for transcriptional responses to IFNs through non-canonical STAT complexes was provided by studies in Stat1^−/−^ mice infected with Sendai virus, MCMV, or DENV, showing that STAT1-independent responses to IFNs provide some level of resistance against these pathogens (83–85). In contrast, the combined loss of IFNγ and type I IFN receptors or of STAT1 and STAT2 results in early death of infected mice (86, 87). Further, consistent with STAT1-independent responses to IFN, Hahm et al. demonstrated that infection of hematopoietic bone marrow cells with MV or LCMV impaired the maturation of dendritic cells in an IFNβ-dependent and Stat2-dependent, but STAT1-independent, manner (88). Additional studies revealed that STAT2 was required for IFN-I-induced expression of a set of ISGs independently of STAT1 (85, 89, 90). Despite the inability to form ISGF3 complexes, Stat1^−/−^ mice and cells could still induce a subset of ISGs in response to the DENV infection (87). This response was absent in compound Stat1/2^−/−^ mice, directing the main attention toward STAT2 as a component of STAT1-independent ISG expression *in vivo*. STAT2 homodimers alone are known to bind DNA very poorly, owing to the lack of a functional DNA-binding domain (91). This suggests that one or more additional components must contribute to STAT1-independent ISG regulation. IRF9, the DNA-binding subunit of the ISGF3 complex is an obvious candidate. The formation of STAT2–IRF9 complexes after IFN-I stimulation had been proposed by several authors based on studies addressing ISG expression in HEK293 cells with overexpressed STAT2 and IRF9 (91), in U3A cells that lack STAT1 (90), and in Hep3B cells (89). In addition, STAT2 was shown to have a STAT1-independent role in the ability of IFNβ and TNFα to synergistically stimulate the expression of Duox2 NADPH oxidase in epithelial cell lines [(92), reviewed in detail in Ref (93, 94); Figure 1]. More recently, the potential of STAT2/IRF9 complexes to stimulate ISG emerged from studies addressing the role of type I IFN in bacterial infections.

As mentioned above, type I IFNs are secreted in response to many bacterial pathogens, such as *Listeria monocytogenes, Francisella tularensis, Legionella pneumophila*, and others (14, 95–97). The impact of IFN-I on bacterial growth in mammalian hosts is variable, as described in detail elsewhere (14). In case of *Legionella pneumophila*, IFN-I inhibits its ability to grow inside macrophages. The growth inhibitory effect was retained in cells isolated from Stat1^−/−^, Stat2^−/−^, or Stat3^−/−^ single knockout animals (17). In contrast, macrophages from compound Stat1/2^−/−^ macrophages were not able to limit the bacterial growth after IFN-I stimulation and had higher bacterial loads than single knockouts of STAT1 or STAT2, pinpointing redundant functions of STAT1 and STAT2 in defense against this bacterial pathogen (98). Studies addressing the molecular mechanism of STAT1-independent ISG expression showed that IFN-I stimulated a delayed activation of STAT2 in Stat1^−/−^ cells. Together with IRF9, STAT2 formed a complex able to bind to the ISRE sequence. Data in Stat1^−/−^ macrophages further demonstrated prolonged JAK activation by the IFN-I receptor and a slow but steady accumulation of both STAT2 and pYSTAT2. The data were consistent with a model according to which a threshold of STAT2 phosphorylation needs to be overcome for the formation of transcriptionally active STAT2–IRF9 complexes and for delayed stimulation of ISG transcription (98, 99). This hypothesis has subsequently been validated in cells derived from mice expressing a STAT1Y701F mutant (99). It is further consistent with reports showing that STAT2 constitutively associates with IRF9 (100) and that this preassociation may be required for rapid generation of the ISGF3 in response to IFN signals in wt cells (101). All the findings together strongly suggest that STAT2–IRF9-stimulated ISG expression could serve as a backup or a support mechanism of defense against pathogens that impede STAT1 signaling or serve to integrate the responses to IFN-I and TNFα (17, 84, 87, 92, 98).

## Novel Aspects of IFNγ Signaling by Canonical and Non-Canonical STAT Complexes

Canonical signaling by the IFNγ receptor causes the formation of GAF, the STAT1 homodimer. GAF activates gene transcription by associating with its cognate DNA-binding sequence, the GAS (2, 102). As in the case of IFN-γ, the reality of transcriptional responses to IFNγ adds complexity. In part, this is due to STAT1 modification. The implications of Y701 phosphorylation as a dimerization signal are undisputed. Similarly, the enhancement of IFNγ-induced gene expression through phosphorylation of the C-terminal S727 and increased association with histone acetylase complexes are well documented (103, 104). Sumoylation of K703 was first described by the group of Curt Horvath (105). The implications of this modification for STAT1 activation and activity were initially unclear (105, 106), but subsequent studies, particularly those in cells derived from mice expressing SUMOylation-defective STAT1, clearly linked SUMOylation to decreased IFNγ responsiveness (107, 108). Mechanistically, SUMOylation interferes with the conjugation of a phosphate at the proximal Y701 and increases nuclear tyrosine dephosphorylation by increasing STAT1’s solubility. In absence of the SUMO modification, STAT1 molecules assemble into an insoluble, phosphatase-resistant paracrystalline array (108, 109). Other than modification, the ability of STAT1 dimers to interact on DNA is essential for the expression of a large fraction of IFNγ-induced genes. This surprising finding emerged from studies in cells and mice expressing a STAT1F77A mutation that inhibits polymerization of promoter-bound STAT1 dimers (110). Strikingly, responsiveness to type I IFN, hence the activity of the ISGF3 complex, was unaffected by the STAT1F77A mutation.

Further variety is introduced to the IFNγ pathway by association between STAT1 and other proteins, i.e., non-canonical complexes (Figure 1). Already in the 1990s, Ifit2, a classical ISRE-regulated gene, was found to be induced by IFNγ in a STAT1- and IRF9-dependent, but STAT2-independent manner (111), suggesting that transcription factors containing both STAT1 and IRF9 are able to control IFNγ-responsive genes. Affirmative observations were made for the expression of the Cxcl10 gene in IFNγ-stimulated 2fTGH cells (112). More recently, work from our lab identified an important role for STAT1/IRF9 in the context of a murine colitis model (113). The Cxcl10 gene, known to contribute to colitogenic inflammation, was shown to be induced downstream of the IFNγ receptor in a STAT1/IRF9-dependent fashion, but independently of STAT2. Molecular analysis confirmed that STAT1/IRF9 complexes form in response to IFNγ and associate with ISRE sequences of enhancer regions 1 and 2 of the Cxcl10 gene promoter.

**Figure 2:**
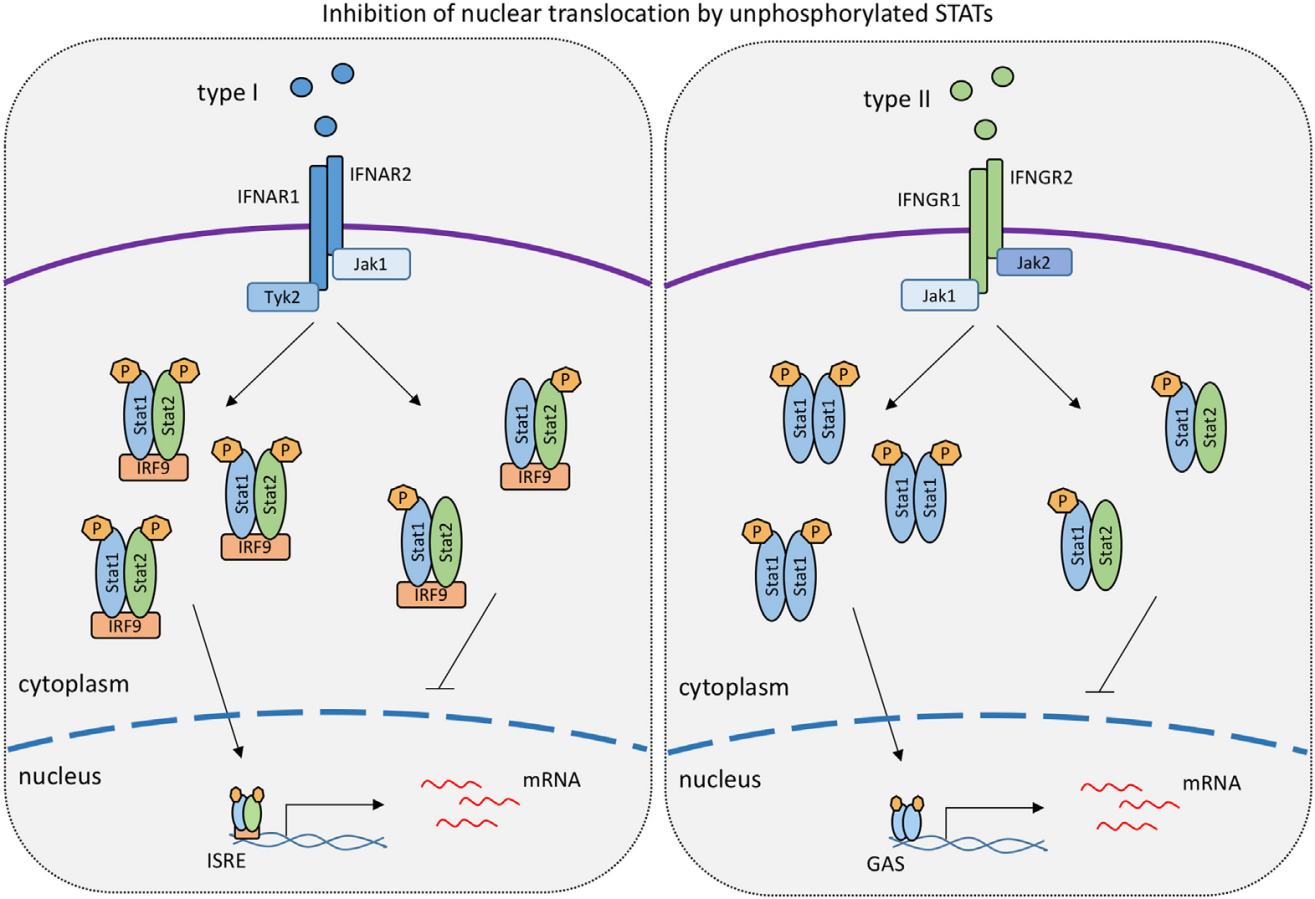
**Levels of unphosphorylated signal transducer and activator of transcriptions (STATs) determine the strength of responses to type I IFN and IFNγ**. The model is based on work published in references 99 and 118 showing that unphosphorylated STAT1 binds to tyrosine-phosphorylated STAT2 and vice versa. Such hemiphosphorylated STAT dimers are incapable of nuclear translocation (118). In the IFNγ response, unphosphorylated STAT2 thus lowers the formation and nuclear translocation of tyrosine-phoshorylated STAT1 dimers (118). Conversely, unphosphorylated STAT1 inhibits the nuclear translocation of tyrosine-phosphorylated STAT2 in the type I IFN response (99).

Other than STAT1/IRF9, STAT2 was proposed to contribute to IFNγ-induced transcription (Figure 1). The extent to which this occurs is unclear, owing in part to the fact that the lack of STAT2 reduces STAT1 levels in some cell types, resulting in a mixed Stat1/Stat2^−/−^ phenotype. For example, Stat2^−/−^ fibroblasts express little STAT1 and show impaired inhibition of vesicular stomatitis virus replication when treated with IFNγ (114). In support of a direct role for STAT2 in the IFNγ response, its tyrosine phosphorylation was reported in a study using IFNγ-treated wild-type mouse primary embryonic fibroblasts. This caused the formation of the ISGF3 transcription factor (115). Similar observations were made by Zimmerman and colleagues in MEFs (116). Stimulation of human lung epithelial cells with IFNγ triggered early and delayed peaks of STAT1 Y701 phosphorylation (117). The delayed peak corresponded to the formation of an ISGF3 complex, named ISGF3^II^, that contained Y701-phosphorylated STAT1 and IRF9, in addition to STAT2 that remained unphosphorylated on Y690. The idea of a STAT2 contribution to the delayed IFNγ response is in line with our recent finding that expression of the Cxcl10 gene is decreased specifically at later stages of the IFNγ response in Stat2^−/−^ macrophages (113). Consistently, we found STAT2 in association with the two Cxcl10 promoter ISREs at the delayed stage of the IFNγ response of wild-type macrophages. Of note, the same study shows that some genes with ISGF3 binding sites did not respond at any time to IFNγ in Stat2^−/−^ macrophages. It appears possible, therefore, that both ISGF3 and ISGF3^II^ complexes contribute to IFNγ-induced transcription in a gene- and stage-specific manner. Of note, however, a stimulatory activity of ISGF3 and ISGF3^II^ complexes is challenged by an entirely different perspective on the role of STAT2 (118). Ho and colleagues show a strong, N-terminal association between unphosphorylated STAT2 and STAT1. This association persists during the IFNγ response and prevents a fraction of STAT1 molecules to enter the nucleus (Figure 2). Consistently, mutations disrupting the N-terminal contacts increase the transcriptional IFNγ response. The study thus presents STAT2 as a moderator of IFNγ-activated STAT1. All findings together support both positive and negative regulation of IFNγ-induced transcription by STAT2. Our studies addressing the Cxcl10 gene suggest that this dual function of STAT2 may represent early and late phases of the transcriptional response to IFNγ, with initial repression being followed by stimulatory activity (110).

## U-STAT Contribution to the IFN Response

According to the original JAK–STAT paradigm, there is a strict correlation between STAT activity and their tyrosine phosphorylation. Defying this notion, numerous reports have meanwhile assigned important tasks to U-STATs lacking a phosphate at the critical tyrosine residue. U-STAT activities include control of organelle metabolism and function in mitochondria or the Golgi apparatus [STAT1, STAT2, and STAT3 and STAT6 in the biology of mitochondria (119–125); STAT5 in the Golgi apparatus (125, 126)]. U-STAT1 was required for TNF-mediated apoptosis of U3A cells. This activity required the protein to be phosphorylated at the C-terminal S727 (127). To address potential functions of U-STAT1 in the immune system, we and our collaborators generated mice expressing a STAT1Y701F mutant and compared immune responses of these animals with Stat1^−/−^ mice (99). Apart from a modest gain of function in antibacterial immunity described below, a notable difference was observed in NK cells. Whereas STAT1 deficiency led to a severe loss in NK cytotoxicity, this is partially retained in Stat1^Y701F^ mice (128). In contrast, U-STAT1 did not rescue the NK maturation defect observed in Stat1^−/−^ mice. Localization to the NK-target cell interface hints at a potential role of U-STAT1 at the immunological synapse. NK cells also demonstrate a further non-canonical activity of STAT1 related to its second phosphorylation site, the C-terminal S727. This site is a target for both p38MAPK and the S/T kinase CDK8 (103, 129–131). Whereas in macrophages or fibroblasts S727 phosphorylation increases the IFNγ-induced expression of a subset of STAT1 target genes, CDK8-mediated S727 phosphorylation in NK cells restricts their cytotoxicity (132). Speculatively, the synaptic pool of STAT1 may be the relevant target, as Stat1^S727A^ mutation in NK cells had little impact on their gene expression. In summary, NK cells reveal several non-canonical activities not only for STAT1, but, as described above, also for TYK2 (Table 1). The integration of these activities in cellular signaling networks remains a future challenge.

In addition to cytoplasm or organelle-based roles, nuclear functions were reported involving U-STATs as either gene repressors or activators (133). For example, in *Drosophila*, STAT92E has been shown to associate with heterochromatin protein 1 (HP1) in cells lacking JAK activity, thus maintaining the structure of heterochromatin and gene repression (134, 135). Likewise, mammalian U-STAT5A reportedly binds to HP1α, stabilizing the heterochromatin in a similar fashion and repressing the genes involved in cancer development (136). In a more recent publication, mouse U-STAT5 was shown to actively repress the transcriptional program required for megakaryocytic differentiation by preventing the binding of the transcriptional activator EGR, acting as a partial antagonist of biological activity of phosphorylated STAT5 (137). U-STAT3 was shown to compete with IκB for binding to unphosphorylated NFκB, translocating to the nucleus and participating in the activation of a subset of NFκB-dependent genes (138). U-STAT6, in cooperation with p300, was suggested to bind to a consensus STAT6 binding site in the promoter of the Cox-2 gene, regulating its constitutive expression (139). The mechanism by which the unphosphorylated STATs enter the nucleus remains to be explored. It is most likely related to the ability of unphosphorylated STATs to shuttle between nucleus and cytoplasm (140). While the nuclear export rate usually localizes most U-STATs in the cytoplasm at steady state, it is conceivable that some of these are trapped in the nucleus by DNA or chromatin association. Other possibilities include the association with non-STAT transcription factors like IRF1, or direct contact with the nuclear pore complex (141–143).

**Figure 3:**
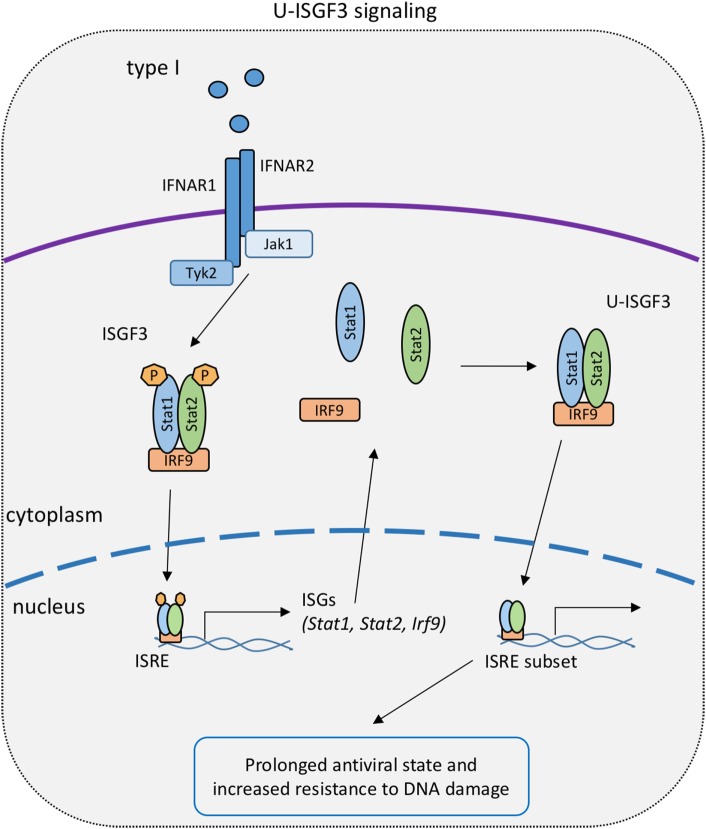
**U-STAT signaling in the type I IFN response**. Early canonical signaling causes the upregulation of IFN-stimulated gene factor 3 (ISGF3) subunits and the subsequent formation of an ISGF3 complex with unphosphorylated signal transducer and activator of transcriptions 1 and 2 [unphosphorylated ISGF3 complex (U-ISGF3)]. U-ISGF3 stimulates a subset of interferon-stimulated genes to prolong the transcriptional response to IFN-I.

The link between U-STATs and the IFN response was made by George Stark and colleagues. The group initially demonstrated an association of U-STAT1 and IRF1 with the partially overlapping interferon consensus sequence 2 and GAS sites in the promoter of the ISG Lmp2. This constitutive interaction resulted in expression of the Lmp2 gene in U3A cells (141). Additional studies, performed in cells overexpressing subunits of the ISGF3 complex, support the concept that U-STATs prolong the expression of a distinct subset of ISG (144) (Figure 3). The authors hypothesize that this occurs as a result of the accumulation of newly synthesized STAT1 and STAT2 following early, canonical type I IFN signaling. According to this hypothesis, prolonged exposure of cells to IFN-I and accumulation of STAT1, STAT2, and IRF9 causes formation of an unphosphorylated ISGF3 complex (U-ISGF3), which in turn maintains the expression of a subset of ISGs that increase resistance to viruses and DNA damage (145).

We attempted to test the U-STAT1 model in Stat1^Y701F^ mice (99). Indeed, some gain of function was noted when cells and animals expressing mutant STAT1 were infected with the bacterial pathogen *Listeria monocytogenes* and compared to STAT1-deficient counterparts. However, Starks U-STAT model could not be tested in these mice owing to the lack of an early, phosphotyrosine-based response to IFN-I that causes an increase of ISGF3 components (Figure 3). In fact, Stat1^Y701F^ mutation caused a drastic decrease in basal levels of STAT1 in cells and animals, due to the lack of tonic signaling by the IFN-I receptor (99, 146). Thus, improved animal models expressing increased U-STAT amounts are needed to collect *in vivo* evidence for their function.

Of interest, the low amounts of U-STAT1 expressed in Stat1^Y701F^ mice acted as suppressors of the delayed STAT1-independent, STAT2-dependent expression of ISGs after IFNβ stimulation (see above). This was a consequence of preventing cytoplasmic, tyrosine-phosphorylated STAT2 from entering the nucleus. The data suggest that hemiphosphorylated STAT dimers do not show the enhanced nuclear translocation of the fully phosphorylated dimers. This notion is in line with the above-mentioned data from the Vinkemeier lab that demonstrate the inability of hemiphosphorylated dimers of wild-type STATs to enter the cell nucleus (118). Therefore, relative ratios of STAT1 and STAT2 and their phosphorylated isoforms may be an important determinant of nuclear signaling by the IFN receptors (Figure 2).

## Functions of IRF9 Beyond IFN Signaling

The studies described above assign an important function of IRF9 to both IFN-I and IFNγ signaling. In this paragraph, we briefly describe some observations linking IRF9 to different diseases, either protecting from or exacerbating pathology. Notably, although IRF9 is an immune regulator, these data demonstrate additional mechanisms that may either link the immune system with these diseases or reflect IRF9 activities unrelated to the immune system. In most cases, the link to IFN signaling remains to be determined. The studies raise the possibility that IRF9 is capable of interacting with other transcription factors to fulfill a different set of functions (147).

Overexpression of IRF9 has been observed in breast and uterine tumors, where it provides resistance to microtubule-disrupting agents through transcriptional activation of ISGs in a STAT1- and STAT2-independent manner (148). In this situation, the ability of IL-6 to act as an inducer of IRF9 may be of importance, as shown for human prostate cancers (149). The role of IRF proteins in adipocyte biology connects the immune response with metabolic regulation (150, 151). In obese mice IRF7 is increased (152), while the expression of IRF3 (153) and IRF9 (152) is decreased. Thus, IRFs respond differently to overnutrition stress. In line with this, mice lacking IRF7 show improved hepatic insulin sensitivity and protection from local and systemic inflammation during high-fat diet (152). In contrast, IRF3 and IRF9 play a protective role in high-fat diet-induced obesity (153, 154). IRF9 was shown to interact with peroxisome proliferator-activated receptor α to regulate gene expression in the liver (154). Their target genes are mostly involved in lipid metabolism, thus attenuating insulin resistance in obese mice. This observation suggests a key role for IRF9 in metabolic functions.

Cardiac hypertrophy and pathological remodeling are hallmarks of cardiomyopathy associated with many pathological stressors. Recent reports found that IRF3, IRF7, and IRF9 protect against cardiac hypertrophy (155–157). In murine disease models, IRF9 binds myocardin, an activator of the transcription factor serum response factor (SRF), thereby inhibiting SRF activation and associated proliferative response. Consistently, an aggravated cardiac hypertrophy occurs in Irf9^−/−^ mice (157). Contrasting its protective effect in cardiac hypertrophy, upregulation of IRF9 during myocardial ischemia–reperfusion (I/R) injury contributes to cardiomyocyte death and inflammation through the Sirt1–p53 axis. In this context, the downregulation of the deacetylase SIRT1 by IRF9 promotes apoptotic signaling through p53 (158). Similarly, IRF9 overexpression leads to cell death signaling in neurons in context of a cerebral ischemic stroke. Hence, IRF9 deficiency mitigates neurological deficits upon stroke (159). In arteria, IRF9 mediates neointima formation, a scar that forms upon vascular injury. IRF9 overexpression increases, and its deficiency decreases the proliferation and migration of vascular smooth muscle cells (VSMCs). As in case of cardiomyocytes, IRF9 suppresses SIRT1 by directly binding to an ISRE in the SIRT1 promoter. Thereby, IRF9 prevents the suppression of AP-1 transactivation by SIRT1. AP-1 induces a vascular injury response pathway that promotes VSMC proliferation in the context of neointima formation (160). Although critical roles of IRF9 in immunity, metabolism, and disease have been revealed, many questions regarding the mechanisms by which IRF9 interconnects such a variety of pathways still remain.

## Concluding Remarks

The overwhelming biological impact of canonical JAK–STAT pathways is undisputed. However, as happens often in biological sciences, long shadows of paradigmatic signaling systems obscure alternative installations of their components for distinct purposes. Recent years of JAK–STAT research have begun to uncover some of these undogmatic events and establish them as non-canonical pathways side-by-side with the canonical ones. This provides food for thoughts about the evolution of JAK–STAT pathways, the emergence of non-canonical and canonical functions. Studies in *Dictyostelium* suggest that STATs evolved without the necessity for tyrosine phosphorylation or the ability to activate transcription (161). Thus, it is tempting to speculate that what we now perceive as a deviation from the canonical pathways is in reality closer to the primordial STAT function that had nothing to do with cytokines or the genes they activate. Although this review provides a very brief overview, we hope it allows readers to get an idea of the many different ways by which non-canonical JAK–STAT pathways are established in the mammalian immune system. Their analysis is far from easy, as results from straightforward experimental approaches are likely to be dominated by the pathway’s canonical output. In spite of this, future research with an open eye for the unexpected holds the promise of new fascinating insights into the many facets of JAKs and STATs in our immune system.

## Author Contributions

AM, EP, EK-H, FR, MM, and TD wrote the review. AM compiled graphics and table.

## Conflict of Interest Statement

The authors declare that the research was conducted in the absence of any commercial or financial relationships that could be construed as a potential conflict of interest.
